# Development and Validation of a Risk Score for Chronic Kidney Disease in HIV Infection Using Prospective Cohort Data from the D:A:D Study

**DOI:** 10.1371/journal.pmed.1001809

**Published:** 2015-03-31

**Authors:** Amanda Mocroft, Jens D. Lundgren, Michael Ross, Matthew Law, Peter Reiss, Ole Kirk, Colette Smith, Deborah Wentworth, Jacqueline Neuhaus, Christoph A. Fux, Olivier Moranne, Phillipe Morlat, Margaret A. Johnson, Lene Ryom, D:A:D study group, the Royal Free Hospital Clinic Cohort, and the INSIGHT, SMART, and ESPRIT study groups

**Affiliations:** 1 Department of Infection and Population Health, University College London, London, United Kingdom; 2 Copenhagen HIV Programme, Department of Infectious Diseases, Rigshospitalet, University of Copenhagen, Copenhagen, Denmark; 3 Division of Nephrology, Mount Sinai School of Medicine, New York, New York, United States of America; 4 The Kirby Institute, University of New South Wales, Sydney, New South Wales, Australia; 5 Division of Infectious Diseases and Department of Global Health, Academic Medical Center, University of Amsterdam, Amsterdam, The Netherlands; 6 University of Minnesota, Minneapolis, Minnesota, United States of America; 7 Clinic for Infectious Diseases and Hospital Hygiene, Kantonsspital Aarau, Aarau, Switzerland; 8 Nephrology Department, Public Health Department, Centre Hospitalier Universitaire de Nice, Nice, France; 9 Université de Bordeaux, INSERM U 897, *Centre Hospitalier Universitaire* de Bordeaux, Bordeaux, France; 10 Department of HIV Medicine, Royal Free London NHS Foundation Trust, London, United Kingdom; Duke University Medical Center, UNITED STATES

## Abstract

**Background:**

Chronic kidney disease (CKD) is a major health issue for HIV-positive individuals, associated with increased morbidity and mortality. Development and implementation of a risk score model for CKD would allow comparison of the risks and benefits of adding potentially nephrotoxic antiretrovirals to a treatment regimen and would identify those at greatest risk of CKD. The aims of this study were to develop a simple, externally validated, and widely applicable long-term risk score model for CKD in HIV-positive individuals that can guide decision making in clinical practice.

**Methods and Findings:**

A total of 17,954 HIV-positive individuals from the Data Collection on Adverse Events of Anti-HIV Drugs (D:A:D) study with ≥3 estimated glomerular filtration rate (eGFR) values after 1 January 2004 were included. Baseline was defined as the first eGFR > 60 ml/min/1.73 m2 after 1 January 2004; individuals with exposure to tenofovir, atazanavir, atazanavir/ritonavir, lopinavir/ritonavir, other boosted protease inhibitors before baseline were excluded. CKD was defined as confirmed (>3 mo apart) eGFR ≤ 60 ml/min/1.73 m^2^. Poisson regression was used to develop a risk score, externally validated on two independent cohorts.

In the D:A:D study, 641 individuals developed CKD during 103,185 person-years of follow-up (PYFU; incidence 6.2/1,000 PYFU, 95% CI 5.7–6.7; median follow-up 6.1 y, range 0.3–9.1 y). Older age, intravenous drug use, hepatitis C coinfection, lower baseline eGFR, female gender, lower CD4 count nadir, hypertension, diabetes, and cardiovascular disease (CVD) predicted CKD. The adjusted incidence rate ratios of these nine categorical variables were scaled and summed to create the risk score. The median risk score at baseline was −2 (interquartile range –4 to 2). There was a 1:393 chance of developing CKD in the next 5 y in the low risk group (risk score < 0, 33 events), rising to 1:47 and 1:6 in the medium (risk score 0–4, 103 events) and high risk groups (risk score ≥ 5, 505 events), respectively. Number needed to harm (NNTH) at 5 y when starting unboosted atazanavir or lopinavir/ritonavir among those with a low risk score was 1,702 (95% CI 1,166–3,367); NNTH was 202 (95% CI 159–278) and 21 (95% CI 19–23), respectively, for those with a medium and high risk score. NNTH was 739 (95% CI 506–1462), 88 (95% CI 69–121), and 9 (95% CI 8–10) for those with a low, medium, and high risk score, respectively, starting tenofovir, atazanavir/ritonavir, or another boosted protease inhibitor.

The Royal Free Hospital Clinic Cohort included 2,548 individuals, of whom 94 individuals developed CKD (3.7%) during 18,376 PYFU (median follow-up 7.4 y, range 0.3–12.7 y). Of 2,013 individuals included from the SMART/ESPRIT control arms, 32 individuals developed CKD (1.6%) during 8,452 PYFU (median follow-up 4.1 y, range 0.6–8.1 y). External validation showed that the risk score predicted well in these cohorts. Limitations of this study included limited data on race and no information on proteinuria.

**Conclusions:**

Both traditional and HIV-related risk factors were predictive of CKD. These factors were used to develop a risk score for CKD in HIV infection, externally validated, that has direct clinical relevance for patients and clinicians to weigh the benefits of certain antiretrovirals against the risk of CKD and to identify those at greatest risk of CKD.

## Editors’ Summary

**Background:**

About 35 million people are currently infected with HIV, the virus that causes AIDS. HIV destroys CD4 lymphocytes and other immune system cells, leaving infected individuals susceptible to other infections. HIV infection can be controlled, but not cured, using combination antiretroviral therapy (cART), and, nowadays, the life expectancy of many HIV-positive individuals is similar to that of HIV-negative people. HIV-positive individuals nevertheless experience some illnesses more frequently than HIV-negative people do. For example, up to a third of HIV-positive individuals develop chronic kidney disease (CKD), which is associated with an increased risk of cardiovascular disease and death. Persons with CKD may have an impaired effect of the filtration units in the kidneys that remove waste products and excess water from the blood to make urine, thereby leading to a reduced blood filtration rate (the estimated glomerular filtration rate [eGFR]) and waste product accumulation in the blood. Symptoms of CKD, which rarely occur until the disease is advanced, include tiredness, swollen feet, and frequent urination. Advanced stages of CKD cannot be cured, but its progression can be slowed by, for example, controlling hypertension (high blood pressure) and diabetes (two CDK risk factors) and by adopting a healthy lifestyle.

**Why Was This Study Done?:**

The burden of CKD may increase among HIV-positive individuals as they age, and clinicians need to know which individuals are at high risk of developing CKD when choosing cART regimens for their patients. In addition, clinicians need to be able to identify those HIV-positive individuals at greatest risk of CKD so that they can monitor them for early signs of kidney disease. Some antiretroviral drugs—for example, tenofovir and atazanavir/ritonavir (a boosted protease inhibitor)—are associated with kidney damage. Clinicians may need to weigh the benefits and risks of giving such potentially nephrotoxic drugs to individuals who already have a high CKD risk. Here, the researchers develop and validate a simple, widely applicable risk score (a risk prediction model) for CKD among HIV-positive individuals and investigate the relationship between CKD and potentially nephrotoxic antiretroviral drugs among individuals with different CKD risk score profiles.

**What Did the Researchers Do and Find?:**

To develop their CKD risk score, the researchers used clinical and demographic data collected from 17,954 HIV-positive individuals enrolled in the Data Collection on Adverse Events of Anti-HIV Drugs (D:A:D) study who had an eGFR > 60 ml/min/1.73 m^2^ and were not taking a potentially nephrotoxic antiretroviral at baseline. During 103,185 person-years of follow-up, 641 individuals developed CKD. Older age, intravenous drug use, hepatitis C coinfection, lower baseline eGFR, female gender, lower CD4 count nadir, hypertension, diabetes, and cardiovascular disease predicted CKD. The researchers included these nine factors in their risk score model (which is available online) and defined three risk groups: low (risk score < 0), medium (risk score 0–4), and high (risk score ≥ 5) risk of CKD development in the next five years. Specifically, there was a 1 in 393, 1 in 47, and 1 in 6 chance of developing CKD in the next five years in the low, medium, and high risk groups, respectively. Because some patients started to use potentially nephrotoxic antiretroviral drugs during follow-up, the researchers were able to use their risk score model to calculate how many patients would have to be treated with one of these drugs for an additional patient to develop CKD over five years in each risk group. This “number needed to harm” (NNTH) for patients starting treatment with tenofovir, atazanavir/ritonavir, or another boosted protease inhibitor was 739, 88, and 9 in the low, medium, and high risk groups, respectively. Finally, the researchers validated the accuracy of their risk score in two independent HIV study groups.

**What Do These Findings Mean?:**

These findings provide a simple, validated risk score for CKD and indicate that the NNTH when starting potentially nephrotoxic antiretrovirals was low among HIV-positive individuals at the highest risk of CKD (i.e., treating just nine individuals with nephrotoxic antiretroviral drugs will likely lead to an additional case of CKD in five years). Although various aspects of the study, including the lack of data on race, limit the accuracy of these findings, these findings highlight the need for monitoring, screening, and chronic disease prevention to minimize the risk of HIV-positive individuals developing diabetes, hypertension, or cardiovascular disease, or becoming coinfected with hepatitis C, all of which contribute to the CKD risk score. Moreover, the development of a tool for estimating an individual’s five-year risk of developing CKD with or without the addition of potentially nephrotoxic antiretroviral drugs will enable clinicians and patients to weigh the benefits of certain antiretroviral drugs against the risk of CKD and make informed decisions about treatment options.

**Additional Information:**

Please access these websites via the online version of this summary at http://dx.doi.org/10.1371/journal.pmed.1001809.

- Information is available from the US National Institute of Allergy and Infectious Diseases on HIV infection and AIDS
- NAM/aidsmap provides basic information about HIV/AIDS, summaries of recent research findings on HIV care and treatment, and personal stories about living with AIDS/HIV
- Information is available from Avert, an international AIDS charity, on many aspects of HIV/AIDS, including personal stories about living with HIV/AIDS
- The World Health Organization provides information on all aspects of HIV/AIDS (in several languages), including its guidelines on the use of ART for treating and preventing HIV infection
- The UNAIDS World AIDS Day Report 2014 provides up-to-date information about the AIDS epidemic and efforts to halt it
- The UK National Health Service Choices website provides information for patients on chronic kidney disease, including some personal stories
- The US National Kidney Foundation, a not-for-profit organization, provides information about chronic kidney disease (in English and Spanish)
- A tool for calculating the CDK risk score developed in this study is available
- Additional information about the D:A:D study is available

## Abstract 3

Amanda Mocroft and colleagues develop and validate a model for determining risk of developing chronic kidney disease for individuals with HIV if treated with different antiretroviral therapies.

## Introduction

HIV infection has become a chronic, manageable infection with a potential life expectancy approaching that of individuals without HIV infection [1,2]. Despite vastly improved outcomes following the introduction of combination antiretroviral therapy (cART) [3], HIV-positive individuals experience increased morbidity, including chronic kidney disease (CKD) [4,5]. The prevalence of CKD in HIV infection has been reported to be as high as 33% [6,7], and is higher in those with both HIV-related risk factors and traditional risk factors, such as diabetes and hypertension [4,8,9]. In addition to immunodeficiency, certain antiretrovirals, including tenofovir, lopinavir/ritonavir, and atazanavir/ritonavir, have also been shown to be associated with chronic renal impairment [9–12].

Deteriorating renal function and CKD are a major health issue for both HIV-positive and-negative individuals, associated with both mortality and cardiovascular outcomes [13–15]. As HIV-positive individuals age, the burden from chronic conditions may increase and identifying those at greatest risk becomes increasingly important. Risk prediction models, or risk scores, have been developed for CKD in both HIV-positive and-negative individuals [16–19]. Such models are not yet widely implemented into routine clinical practice, with concerns about poor study design and lack of external validation of these scores [20]. Implementation of risk score models as part of routine care would allow graded consideration of the safest drugs when initiating and switching antiretrovirals, as well as identifying individuals for whom more intensive renal monitoring may be appropriate.

The aim of this study was to develop a simple, externally validated, and widely applicable long-term risk score model for CKD in HIV-positive individuals that can guide decision making in clinical practice, and to identify those at greatest risk of CKD.

## Methods

### Ethical and Study Approval

All participating cohorts in the Data Collection on Adverse Events of Anti-HIV Drugs (D:A:D) study followed local national guidelines/regulations regarding patient consent and ethical review. Of the countries represented by the participating cohorts, only Switzerland and Australia required specific ethical approval for the D:A:D study in addition to that required for their national cohorts (Swiss HIV Cohort Study and Australian HIV Observational Database). France, Italy, and Belgium did not require specific ethical approval over and above that required for the individual cohorts (Nice/Aquitaine Cohort, Brussels St. Pierre Cohort, and ICONA Cohort, respectively), and the Netherlands did not require any specific ethical approval since the data were provided as part of HIV care (ATHENA Cohort). For the EuroSIDA study (which includes the data from the BASS and Swedish cohorts), each participating site has a contractual obligation to ensure that data collection and sharing is done in accordance with national legislation; each site principal investigator either maintains appropriate documentation from an ethical committee (if required by law) or has a documented written statement to say that this is not required.

Use of data from the Royal Free Hospital Clinic Cohort was approved by the Chairman of the Ethics Committee. Both the SMART and ESPRIT trials were approved by the institutional review board at each participating site, and written informed consent was obtained from all participants. The institutional review board at the University of Minnesota approved the INSIGHT study (0603M83587).

This project and analysis of data was approved by the D:A:D steering committee on 28 November 2013 and further ratified by the renal working group within D:A:D on 29 April 2014. A formal project proposal was submitted to the principle investigator of the Royal Free Hospital Clinic Cohort (M. A. J.) and to the scientific committee of INSIGHT for their approval and release of data. Approval from the Royal Free Hospital Clinic Cohort was received on 21 May 2014, and from the INSIGHT scientific steering committee on 20 May 2014.

### Study Population

The D:A:D study is a prospective cohort collaboration established in 1999 that follows more than 49,000 HIV-1-positive individuals in Europe, the United States, and Australia; details have been published previously [21]. Data on routine clinical care, including demographic factors, antiretroviral therapy (ART), laboratory values, cardiovascular risk factors, and AIDS events, are collected electronically at enrolment and every 6 mo thereafter. Serum creatinine measurements have been collected systematically in participating cohorts since January 2004.

### Patients

Baseline was defined as the first estimated glomerular filtration rate (eGFR) > 60 ml/min/1.73 m^2^ after the later of (1) 1 January 2004 or (2) inclusion into the D:A:D study. eGFR values were calculated using the Cockcroft-Gault formula [22], standardised for body surface area. Individuals were censored at the earlier of (1) last eGFR or (2) end of clinical follow-up, defined as the earlier of (1) last visit plus 6 mo or (2) 1 February 2013. Individuals with <3 eGFR measurements (including the baseline eGFR) before the censor date were excluded, as were individuals with less than 3 mo of follow-up, who could not progress to CKD. CKD was defined as a confirmed (>3 mo apart) decrease in eGFR to ≤60 ml/min/1.73 m^2^ [23]. We excluded individuals with exposure to the potentially nephrotoxic antiretrovirals tenofovir, atazanavir, atazanavir/ritonavir, lopinavir/ritonavir, or other boosted protease inhibitors (any other protease inhibitor used with ritonavir) before baseline. These antiretrovirals have previously been shown to be related to renal outcomes [9,24]. Individuals starting the potentially nephrotoxic antiretrovirals after baseline were included. Individuals with prior exposure to abacavir and indinavir were not excluded, and the relationship with development of CKD was investigated in models adjusting for their use. cART was defined as any regimen containing three or more antiretrovirals from any class. Hepatitis B, hepatitis C, hypertension, diabetes, and anaemia were defined as in previous D:A:D analyses (further information available in [11,25]).

### Statistical Methods

As CKD can develop over many years and HIV-positive individuals are exposed to antiretrovirals for many years, we aimed to develop a risk score for predicting CKD over the medium term (5 y). The first stage was to develop a risk score model to identify the non-antiretroviral factors associated with the development of CKD; the relationship between CKD and the potentially nephrotoxic antiretrovirals was then investigated after adjustment for the demographic and clinical factors associated with CKD found in stage 1.

In stage 1, Poisson regression was used to determine the risk of CKD using information known at baseline. This model was developed in individuals who were not previously exposed to a potentially nephrotoxic antiretroviral, but individuals may have subsequently started one of these drugs. All potential demographic, HIV-related, and cardiovascular risk factors for CKD variables were examined univariately, and factors with *p* < 0.1 were included in a multivariate model. All variables not selected for inclusion (*p* ≥ 0.1) were added to the final model in turn to determine whether their inclusion improved the fit of the model, as evidenced by *p* < 0.1 or a lower Akaike information criterion value. Continuous variables were fitted as continuous linear variables, categorical variables, or continuous non-linear variables with polynomial splines, depending on previously published literature, clinical value, and best fitting models.

As data on a number of the renal risk factors (smoking status, hypertension, diabetes, and prior CVD) are not routinely collected worldwide, a short score was derived without these variables to allow a risk score calculation in those individuals where only basic HIV information is known. The adjusted incidence rate ratios (aIRRs) from the best fitting model were scaled and used to determine the components of the risk score. The components were scaled by dividing all the aIRRs by the smallest and rounding to the nearest whole number. Whole numbers were used rather than the exact aIRRs to allow the risk score to be easily remembered and calculated. Quintiles of the derived risk score were used to define five categories of risk; the lowest three quintiles were combined as the absolute risk in these categories was extremely small, leaving three risk groups, low (risk score < 0), medium (risk score 0–4), and high (risk score ≥ 5). The same categories were used to classify the shortened version of the risk score (short score). Kaplan-Meier methods were used to determine the probability of CKD at 5 y after baseline.

### Derivation of the Best Fitting Model for CKD

Different methods of building the CKD risk score were also investigated to determine whether similar predictors of CKD were identified. Forward selection, i.e., including the most important factors first (those with the lowest *p*-value and/or those with the greatest change in Akaike information criterion), led to the same model choice, as did backward selection, i.e., fitting all potential explanatory variables and removing them one at a time, depending on their importance. A priori, specific interactions of clinical relevance (e.g., age and hypertension) were identified and tested, with appropriate correction for multiple statistical testing. None of the interactions tested were significant, and therefore the interactions were not included in the CKD risk score model.

### Initiation of Potentially Nephrotoxic Antiretrovirals

Initiation of a potentially nephrotoxic antiretroviral was added as a time-dependant covariate to the risk score model, including exactly the same patients contributing the same person-years of follow-up (PYFU), to estimate the possible contribution of potentially nephrotoxic antiretrovirals to CKD development. All individuals were naïve to these drugs at baseline, but could subsequently start during prospective follow-up. The risk score model from stage 1 was used to calculate the number needed to harm (NNTH) over 5 y for those at low, medium, and high risk of CKD. 95% confidence intervals were estimated based on the 95% confidence intervals from the Kaplan-Meier progression rates.

### External Validation of the Risk Score

The risk score was validated using two external cohorts of HIV-positive individuals: the Royal Free Hospital Clinic Cohort [26] and the control arms (i.e., non-intervention) of the SMART and ESPRIT trials from the INSIGHT network [27,28]. Inclusion and exclusion criteria were applied in the same way as for D:A:D study participants; eGFR was calculated using the Chronic Kidney Disease Epidemiology Collaboration (CKD-EPI) formula [29]. Risk score performance was evaluated by comparison of the crude incidence rates within risk score strata, Kaplan-Meier progression rates, and aIRRs associated with a one-point increase in the risk score.

### Calculation of an Individual’s Risk of CKD at *t* Years

For a given set of characteristics, the probability of progression to CKD in the next *t* years (e.g., 5 y) can be calculated using the following formula:

Prob (survival at timet)=1−exp(−H∗t) (1)

where *H* = exp (β_0_ + β_1_X_1_ + β_2_X_2_); X_1_, X_2_, etc., are the covariate values; and β_0,_ β_1_, β_2_, etc., are the parameter estimates from the Poisson regression model.

The population was divided into those at low, moderate, and high risk of CKD for the purpose of explaining the model and to broadly categorise risk of CKD. Individual risk can be calculated as per this formula. The authors will develop an easy-to-use online tool whereby an individual’s underlying risk of CKD and NNTH depending on types of antiretroviral drugs used can be estimated by entering the individual’s own characteristics.

All analyses were performed using SAS (SAS Institute, Cary, North Carolina, US), version 9.3.

## Results

### Included Patients

Of the 49,717 individuals enrolled in the D:A:D study, 36,180 (72.8%) had at least one eGFR > 60 ml/min/1.73 m^2^ after the later of 1 January 2004 or enrolment into the D:A:D study. Of these 36,180 individuals, 17,954 (49.6%) met entry criteria for and were included in analyses; 2,645 (7.3%) individuals were excluded because they had <3 eGFR measurements, 308 (0.9%) were excluded because they had no baseline CD4 counts or viral loads, 15,172 (41.9%) were excluded because of prior exposure to potentially nephrotoxic antiretrovirals, and 101 (0.3%) were excluded because there was <3 mo of follow-up between first eGFR after baseline and last eGFR. The incidence of CKD in those excluded was 12.7/1,000 PYFU (95% CI 12.0–13.5).

The characteristics of the 17,954 included individuals are shown in Table 1. The median eGFR at baseline was 104 (interquartile range [IQR] 90–120 ml/min/1.73 m^2^); 687 individuals (3.8%) had an eGFR of >60 to ≤70 ml/min/1.73 m^2^, 3,778 (21.0%) had an eGFR of >70 to ⩽90 ml/min/1.73 m^2^, and 13,489 (75.1%) had an eGFR of >90 ml/min/1.73 m^2^. There was a total of 270,926 eGFR measurements available for analysis, a median of 14 (IQR 9–20) per person, measured a median time of 4 mo (IQR 3–6) apart. During 103,185 PYFU (median follow-up 6.1 y, range 0.3–9.1 y), 641 developed CKD (3.6%); the incidence of CKD was 6.2/1,000 PYFU (95% CI 5.7–6.7). At 2, 5, and 8 y after baseline, 1.1% (95% CI 0.9%–1.2%), 2.7% (95% CI 2.4%–2.9%), and 5.3% (95% CI 4.9%–5.8%) were estimated to have developed CKD, respectively.

**Table 1:** Baseline characteristics of patient population.

Characteristic	Subcategory	All		Did Not Develop CKD		Developed CKD	
		*N* or Median	Percent or IQR	*N* or Median	Percent or IQR	*N* or Median	Percent or IQR
**All**		17,954	100	17,313	96.4	641	3.6
**Gender**	Male	13,130	73.1	12,671	73.2	459	71.6
	Female	4,824	26.9	4,642	26.8	182	28.4
**Race**	White	8,361	46.6	8,040	46.4	321	50.1
	Black	1,286	7.2	1,264	7.3	22	3.4
	Other	356	2.0	348	2.0	8	1.3
	Unknown	7,951	44.3	7,661	44.3	290	45.2
**HIV exposure**	Homosexual	8,310	46.3	8,046	46.5	264	41.2
	Intravenous drug user	2,081	11.6	1,995	11.5	86	13.4
	Heterosexual	6,578	36.6	6,333	36.6	245	38.2
	Other	985	5.5	939	5.4	46	7.2
**Hepatitis B**	Negative	11,070	61.7	10,699	61.7	371	57.9
	Positive	1,633	9.1	1,574	9.1	59	9.2
	Unknown	5,251	29.2	5,046	29.2	211	32.9
**Hepatitis C**	Negative	11,386	63.4	10,995	63.4	391	61.0
	Positive	2,262	12.6	2,173	12.6	89	13.9
	Unknown	4,306	24.0	4,145	24.0	161	25.1
**Antiretroviral use**	Naïve	9,325	51.9	9,126	52.7	199	31.0
	ART	512	2.9	488	2.8	24	3.7
	cART	8,117	45.2	7,699	44.5	418	65.2
**Smoking status**	Current	7,439	41.4	7,219	41.7	220	34.3
	Previous	3,111	17.3	2,956	17.1	155	24.2
	Never	5,019	28.0	4,812	27.8	207	32.3
	Unknown	2,385	13.3	2,326	13.4	59	9.2
**Hypertension**		1,448	8.1	1,328	7.7	120	18.7
**CVD**		295	1.6	251	1.5	44	6.9
**AIDS**		2,628	14.6	2,469	14.3	159	24.8
**Recent AIDS (in last 12 mo)**		610	23.2	586	23.7	24	15.1
**Diabetes**		562	3.1	494	2.9	68	10.6
**Anaemia^1^**		3,019	22.3	2,852	21.8	167	33.3
**Viral load < 400**		7,913	44.1	7,515	43.4	398	62.1
**Age (years)**		40	34–47	40	33–46	56	47–64
**CD4 count (cells/mm^3^)**		460	320–643	460	320–644	440	300–615
**Nadir CD4 count (cells/mm^3^)**		290	169–434	292	170–438	202	93–337
**Date of baseline eGFR^2^ (month/year)**		6/05	5/04–1/07	6/05	5/04–2/07	1/05	5/04–1/06
**Baseline eGFR^2^ (ml/min/1.73 m^2^)**		104	90–120	105	91–121	73	65–84

^1^Baseline haemoglobin known for 13,568 (75.6%) overall: 13,067 (75.5%) among those who did not develop CKD, and 501 (78.2%) among those who developed CKD.

^2^First eGFR > 60 ml/min/1.73 m^2^ after later of 1 January 2004 or enrolment into the D:A:D study.

### Development of the Risk Score and the Short Version Excluding Cardiovascular Risk Factors

HIV exposure group (intravenous drug user versus all others), gender, hepatitis C coinfection, age, nadir CD4 count (≤200 cells/mm^3^), baseline eGFR, hypertension, diabetes, and prior CVD at baseline were all significant predictors of CKD and were included in the risk score model. Both the precise coefficients from the best fitting statistical model and the estimates to be used in the risk score model are shown in Table 2. Baseline CD4 count, hepatitis B status, race, smoking status, AIDS ever or in the 12 mo prior to baseline, prior use of other antiretrovirals (including abacavir or indinavir), anaemia, and viral load (as either a continuous or categorical variable) were not significantly related to CKD after adjustment for the other variables listed and were excluded from our risk score models.

**Table 2:** Models for risk score for CKD: full risk score and short risk score.

Characteristic	Subcategory	Full Risk Score Model		Example of Calculation for Full Risk Score Model		Short Risk Score Model (Unknown Cardiovascular Risk)	
		Exact Coefficient	Coefficient to Use in Risk Score Calculation	Characteristic	Contribution	Exact Coefficient	Coefficient to Use in Short Risk Score Calculation
**Intercept** ^1^		−6.2406	0			−6.2059	
**HIV exposure**	Not intravenous drug user	0		X	0	0	
	Intravenous drug user	0.6556	2			0.6481	2
**Hepatitis C coinfection**	Negative	0		X	0	0	
	Positive	0.3395	1			0.3288	1
**Age (years)**	≤35	0				0	
	>35 to ≤50	1.0813	4	X	4	1.1114	4
	>50 to ≤60	2.0276	7			2.1166	7
	>60	2.7841	10			2.9537	10
**Baseline eGFR (ml/min/1.73 m^2^)**	>60 to ≤70	1.6475	6	X	6	1.6254	6
	>70 to ≤90	0				0	
	>90	−1.6365	−6			−1.6242	−6
**Gender**	Male	0				0	
	Female	0.3982	1	X	1	0.3701	1
**Nadir CD4 count (cells/mm^3^)**	≤200	0				0	
	>200	−0.2848	−1	X	−1	−0.2931	−1
**Hypertension^2^**	No	0		X	0		
	Yes	0.2386	1				
**Prior CVD^2^**	No	0		X	0		
	Yes	0.4097	1				
**Diabetes^2^**	No	0					
	Yes	0.5764	2	X	2		

Individuals with unknown values for hepatitis, hypertension, prior CVD, and diabetes were included in the “no” category for those factors after comparing the coefficients from the multivariate model. Models excluding those with unknown data showed similar factors associated with CKD, and a similar relative contribution from each of the factors considered.

^1^For use if the exact risk is to be calculated.

^2^See [11] for further information.

Also shown in Table 2 is the shortened version of the risk score, which could be used when cardiovascular risk factors are unavailable. The improvement in event classification by incorporating the cardiovascular risk factors in the risk score model was 2.5% (*p* <0.001). The risk score model had good discrimination (Harrell’s C-statistic 0.92, 95% CI 0.90–0.93), as did the short model (Harrell’s C-statistic 0.91, 95% CI 0.90–0.92), although significantly poorer than the risk score model incorporating cardiovascular risk factors (*p* < 0.001). The short model had a significantly higher Akaike information criterion, indicating that the risk score model provided a better prognostic risk score.

Focusing on the full risk score model, the median risk score at baseline was −2 (IQR −4 to 2). Among those who developed CKD, the median risk score at baseline was 10 (IQR 5–14). Three risk groups were created, as detailed in the Methods, corresponding to low (risk score < 0, 33 events [5.1%]), medium (risk score 0–4, 103 events [16.1%]), and high risk (risk score ≥ 5, 505 events [78.8%]). There was a clear increase in incidence of CKD moving from the low to high risk score group (Table 3), and these rates and proportions reflect the underlying risks of CKD in individuals not exposed to potentially nephrotoxic antiretrovirals; there was a 0.18% probability of developing CKD over the next 5 y in the low risk group (95% CI 0.09%–0.26%), increasing to 1.50% (95% CI 1.09%–1.91%) and 14.68% (95% CI 13.24%–16.12%) in the medium and high risk groups, respectively. Finally, there was a 1:393 chance of developing CKD in the next 5 y in the low risk group, rising to 1:47 and 1:6 in the medium and high risk groups, respectively.

**Table 3:** Comparison of the risk score model in the derivation and validation cohorts.

Outcome	Derivation D:A:D Cohort	Derivation D:A:D Cohort (Short Risk Score)	Validation SMART/ESPRIT	Validation Royal Free Hospital Clinic Cohort (Short Risk Score)
***N***	17,954	17,954	2,013	2,548
**Developed CKD, *N* (percent)**	641 (3.6)	641 (3.6)	32 (1.6)	94 (3.7)
**Incidence of CKD/1,000 PYFU (95% CI)**	6.2 (5.7–6.7)	6.2 (5.7–6.7)	3.8 (2.5–5.1)	5.1 (4.1–6.1)
**Risk score model**				
Baseline score, median (IQR)	−2 (−4 to 2)	−2 (−4 to 2)	−2 (−3 to 1)	−2 (−5 to 0)
Baseline score for those who developed CKD, median (IQR)	10 (5–14)	10 (5–13)	9 (0–12)	5 (1–9)
Events by score group: low/medium/high	33/103/505	37/112/492	6/7/19	18/21/55
**Incidence of CKD/1,000 PYFU (95% CI)**				
Low (risk score < 0)	0.51 (0.34–0.69)	0.56 (0.38–0.75)	1.07 (0.39–2.32)	1.32 (0.78–2.08)
Medium (risk score 0–4)	4.27 (3.45–5.10)	4.67 (3.80–5.53)	3.40 (1.35–6.92)	6.55 (3.75–9.35)
High (risk score ≥ 5)	34.75 (31.72–37.78)	36.05 (32.86–39.23)	25.90 (15.60–40.50)	36.33 (26.73–45.93)
**Incidence rate ratio (95% CI)**				
Low (risk score < 0)	0.12 (0.081–0.18)	0.12 (0.08–0.18)	0.32 (0.11–0.94)	0.20 (0.11–0.38)
Medium (risk score 0–4)	1	1	1	1
High (risk score ≥ 5)	8.13 (6.58–10.05)	7.73 (6.29–9.49)	7.71 (3.24–18.35)	5.55 (3.36–9.18)
**Kaplan-Meier percent progressed at 5 y (95% CI)**				
Low (risk score < 0)	0.18 (0.09–0.26)	0.19 (0.10–0.27)	0.38 (0.10–0.81)	0.35 (0.04–0.66)
Medium (risk score 0–4)	1.50 (1.09–1.91)	1.62 (1.19–2.05)	1.64 (0.15–3.13)	1.71 (0.34–3.08)
High (risk score ≥ 5)	14.68 (13.24–16.12)	15.33 (13.82–16.84)	11.30 (5.62–16.98)	15.00 (9.90–20.10)
**aIRR (95% CI) per unit increase in score**	1.32 (1.30–1.34)	1.33 (1.31–1.34)	1.30 (1.23–1.37)	1.29 (1.25–1.33)

### Validation of the Risk Score in Two External Cohorts

There were 55,010 eGFR measurements included from the Royal Free Hospital Clinic Cohort from 2,548 individuals; 94 individuals developed CKD (3.7%) during 18,376.0 PYFU (incidence 5.1/1,000 PYFU; 95% CI 4.1–6.1; median follow-up 7.4 y, range 0.3–12.7 y). Characteristics are shown in S1 Table. Information on diabetes, CVD, and hypertension was not routinely recorded in this cohort, and hence the short risk score was validated, as summarised in Table 3.

There were 11,088 eGFR measurements included from the SMART/ESPRIT trials from 2,013 individuals; 1,036 (51.5%) were from ESPRIT. In total, 32 individuals developed CKD (1.6%) during 8,451.7 PYFU (incidence 4.1/1,000 PYFU, 95% CI 2.2–5.9; median follow-up 4.1 y, range 0.6–8.1 y). Cohort characteristics are shown in S1 Table. Information on diabetes, hypertension, and prior CVD was available within the SMART/ESPRIT cohorts, and hence the full risk score was validated (Table 3).

**Fig 1:**
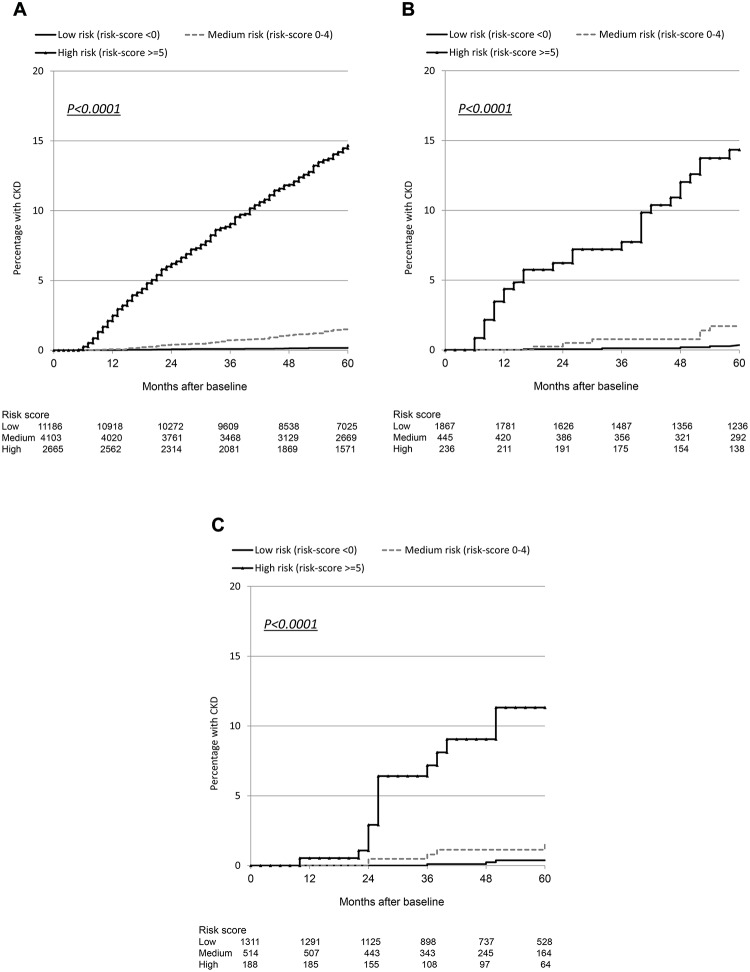
Kaplan Meier progression to chronic kidney disease with low, medium, and high risk scores. (A) D:A:D study: derivation cohort. (B) Royal Free Hospital Clinic Cohort: validation cohort. (C) SMART/ESPRIT control arms: validation cohort.

Although the number of events was considerably smaller in the validation cohorts than in the derivation cohort, resulting in comparatively wide 95% confidence intervals, the incidence rates were similar and increased across the low, medium, and high risk score groups (Table 3). Incidence rates across derivation and validation cohorts were similar, and the aIRR associated with a one-point increase in the score was virtually identical in all cohorts, using the full risk score or the short version. Kaplan-Meier progression in those with low, medium, and high risk scores was also very similar in both of the validation cohorts and the D:A:D derivation cohort (Fig. 1; Table 3). The short risk score showed good discrimination in the Royal Free Hospital Clinic Cohort (Harrell’s C-statistic 0.86, 95% CI 0.78–0.90), as did the full risk score in the SMART/ESPRIT cohort (Harrell’s C-statistic 0.87, 95% CI 0.80–0.94).

### Starting Potentially Nephrotoxic Antiretrovirals and Number Needed to Harm

Table 4 shows the initiation of antiretrovirals after baseline. For example, 11,153 individuals started tenofovir during prospective follow-up (62.1%), and 9,854 had started the drug by 5 y after baseline, corresponding to 61.2% (95% CI 60.4%–62.0%) starting tenofovir by 5 y using Kaplan-Meier estimation. Initiation of a potentially nephrotoxic antiretroviral was added to the risk score model as a time-dependant covariate to estimate contribution of potentially nephrotoxic antivirals to the risk of CKD and to calculate the NNTH for those at low, medium, and high risk of CKD. Individuals starting tenofovir, atazanavir/ritonavir, or any other boosted protease inhibitor (excluding lopinavir/ritonavir) had an increased risk of CKD equivalent to a two-point increase in the risk score, or a 74% increased incidence of CKD (aIRR 1.74, 95% CI 1.69–1.78) compared to individuals not starting these drugs but with otherwise similar risk factors for CKD. Those starting atazanavir or lopinavir/ritonavir had an increased risk of CKD equivalent to a one-point increase in the risk score, or a 32% increased incidence of CKD (aIRR 1.32, 95% CI 1.30–1.34) compared to those not starting these drugs but with otherwise similar risk factors for CKD.

**Table 4:** Use of potentially nephrotoxic antiretrovirals during follow-up in among 17,954 individuals in the D:A:D study.

Outcome	Tenofovir	Atazanavir	Atazanavir/r	Lopinavir/r	Other PI/r	Any of These
**Started ARV, *N* (percent)**						
After baseline	11,153 (62.1)	1,078 (6.0)	3,441 (19.2)	3,240 (18.1)	440 (2.5)	12,641 (70.4)
By 1 y	3,668 (20.4)	316 (1.8)	992 (5.5)	898 (5.0)	96 (0.5)	4,448 (24.8)
By 2 y	5,655 (31.5)	497 (2.8)	1,628 (9.1)	1,445 (8.0)	183 (1.0)	6,988 (38.9)
By 5 y	9,854 (54.9)	799 (4.5)	2,744 (15.3)	2,515 (14.0)	317 (1.8)	11,474 (63.9)
**Developed CKD and started ARV after baseline, *N* (percent)**	366 (57.1)	46 (7.2)	162 (25.3)	128 (20.0)	16 (2.5)	434 (67.7)
**Kaplan-Meier percentage started ARV, percent (95% CI)**						
At 1 y	19.3 (18.7–19.9)	0.8 (0.7–1.0)	5.0 (4.7–5.3)	7.6 (7.2–8.0)	0.7 (0.6–0.8)	24.9 (24.2–25.5)
At 2 y	32.3 (31.6–33.0)	1.8 (1.6–2.0)	9.4 (9.0–9.8)	11.4 (10.9–11.8)	1.3 (1.1–1.4)	39.8 (39.1–40.6)
At 5 y	61.2 (60.4–62.0)	5.2 (4.8–5.5)	18.7 (18.1–19.3)	18.7 (18.1–19.3)	2.5 (2.3–2.8)	70.6 (69.8–71.3)

All individuals were naïve to these antiretrovirals at baseline.

/r, ritonavir boosted; ARV, antiretroviral; PI, protease inhibitor.

**Fig 2:**
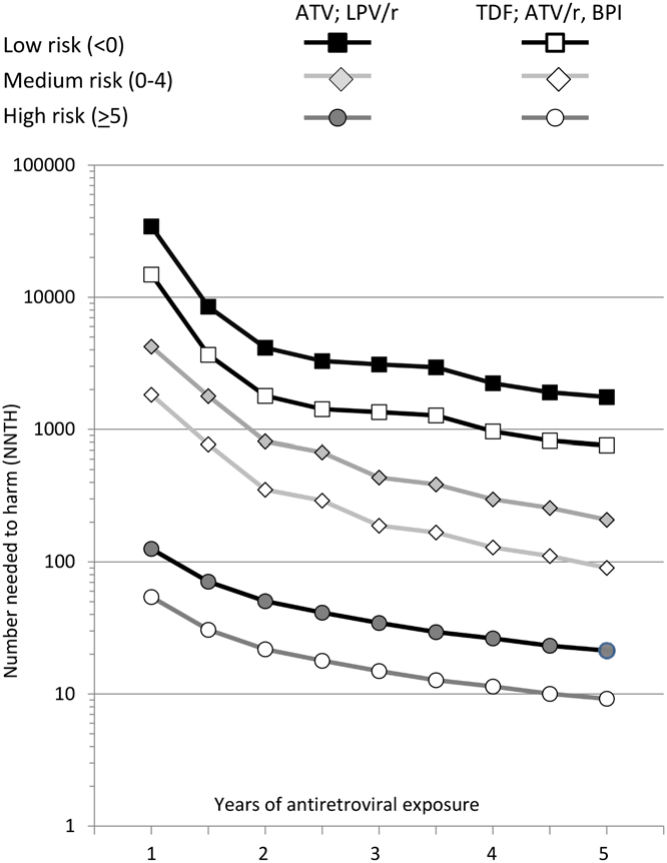
Number needed to harm among those at low (risk score < 0), medium (risk score 0–4), or high risk (risk score ≥ 5) of CKD. ATV, atazanavir; ATV/r, atazanavir/ritonavir; BPI, other ritonavir-boosted protease inhibitor (excluding lopinavir/ritonavir and atazanavir/ritonavir); LPV/r, lopinavir/ritonavir; TDF, tenofovir.

NNTH values are summarised in Fig. 2. The NNTH is the average number of individuals who could be treated with one of the potentially nephrotoxic antiretrovirals for one extra person to develop CKD compared to if the same individuals had not received this treatment. There is a clear decrease in NNTH moving from low to high risk score group for CKD, suggesting much greater potential harm with these potentially nephrotoxic antiretrovirals in individuals at high risk of CKD compared to those at low risk. For example, starting or adding tenofovir, atazanavir/ritonavir, or another boosted protease inhibitor (excluding lopinavir/ritonavir) gave a NNTH of 739 over 5 y in those at low risk of CKD. Thus, on average, treating 739 individuals (95% CI 506–1,462) with tenofovir in the low risk group would yield one extra case of CKD relative to if the same 739 individuals were not treated with this drug. Starting or adding unboosted atazanavir or lopinavir/ritonavir in the low risk group gave a NNTH of 1,702 over 5 y (95% CI 1,166–3,367). In the medium risk group (risk score 0–4), the NNTH was 88 (95% CI 69–121) for tenofovir, atazanavir/ritonavir, or another boosted protease inhibitor (excluding lopinavir/ritonavir) and 202 (95% CI 159–278) for unboosted atazanavir or lopinavir/ritonavir, over 5 y. These NNTH values decreased markedly in the high risk group (risk score ≥ 5), to 9 (95% CI 8–10) and 21 (95% CI 19–23), respectively, over 5 y.

The NNTH is for addition of each potentially nephrotoxic antiretroviral as a single drug; in practice they are commonly combined. For example, a person starting tenofovir with lopinavir/ritonavir would have a 2.29-fold increased incidence of CKD (1.74—fold increase from tenofovir and 1.32-fold increase from lopinavir/ritonavir), equivalent to a three-point increase in the risk score. This equates to a NNTH of 436 (95% CI 298–861) for those at low risk, 52 (95% CI 41–71) for those at medium risk, and 5 (95% CI 5–7) for those at high risk.

### Calculation of an Individual Risk Score

Using Table 3, a person who is female (+1), who does not use intravenous drugs (+0), without hepatitis coinfection (+0), aged 38 y (+4), who has an eGFR of 68 ml/min/1.73 m^2^ (+6), with a nadir CD4 count of 250 cells/mm^3^ (−1), without hypertension (+0) or prior CVD (+0), but with diabetes (+2) would have a risk score of 12, placing her in the high risk category. On average, 14.68% (95% CI 13.24%–16.12%) of individuals in this study with a high risk of CKD progressed to CKD by 5 y after baseline.

The exact coefficients can also be used to determine an individual’s risk, where the person’s characteristics can be used to calculate the exact probability of the risk of CKD at 5 y and estimate how that risk changes if one of the potentially nephrotoxic antiretrovirals is started. Thus a person who is female (+0.3982), who does not use intravenous drugs (+0), without hepatitis coinfection (+0), aged 38 y (+1.0813), who has an eGFR of 68 ml/min/1.73 m^2^ (+1.6475), with a nadir CD4 count of 250 cells/mm^3^ (−0.2848), without hypertension (+0) or prior CVD (+0), but with diabetes (+0.5764) would have an exact risk score of 3.4186, added to the constant (representing the intercept of the model) of −6.2406, giving a total score (*H*) of −2.822, corresponding to a 25.7% risk of developing CKD in 5 y; adding tenofovir would increase that risk to 44.7% (i.e., a 74% increase in risk of CKD in those starting tenofovir); adding tenofovir and lopinavir/ritonavir would increase the risk to 58.9% (i.e., a 123% increase in risk). In contrast, a person who is male (+0), who does not use intravenous drugs (+0), without hepatitis coinfection (+0), aged 28 y (+0), who has an eGFR of 80 ml/min/1.73 m^2^ (+0), with a nadir CD4 count of 250 cells/mm^3^ (−0.2848), without hypertension (+0), prior CVD (+0), or diabetes (+0) would have an exact risk score of −0.2848, added to the constant (representing the intercept) of −6.2406, giving a total score (*H*) of −6.5254, corresponding to a 0.7% risk of developing CKD within the next 5 y, increasing to 1.2% if tenofovir is included as part of his antiretroviral regimen, and to 1.6% if he starts tenofovir and lopinavir/ritonavir.

## Discussion

We developed a simple, clinically useful risk score for predicting CKD at 5 y in individuals with HIV, and validated the score in two clinically diverse populations with different frequencies of eGFR monitoring. We have calculated the NNTH over 5 y when starting tenofovir or other potentially nephrotoxic antiretrovirals in individuals with high, medium, or low underlying risk of CKD. The development of a publicly available online tool estimating an individual’s 5-y risk of CKD with or without the addition of potentially nephrotoxic antiretrovirals enables clinicians and HIV-positive individuals to make an informed decision about acceptable risk for an individual’s care, relative to the benefits of any given treatment regimen, and to identify those at greatest risk of CKD.

Previous research from the D:A:D study showed similar risk factors for end-stage renal disease/advanced CKD, and showed that those on tenofovir had lower rates of this more advanced end point [30]. The most likely reason for this finding was that tenofovir, especially, was discontinued as eGFR declined, likely leaving a highly selected group of individuals at low risk of end-stage renal disease taking the drug. The previous study differs from that presented here in two important ways: by considering a less advanced deterioration in kidney function than CKD, and by considering the medium- to long-term risk of CKD to develop a risk score. Age, HIV exposure route, hepatitis C coinfection, gender, nadir CD4 count, hypertension, CVD, and diabetes, have all been previously shown to be associated with CKD [4,19,31], so their inclusion in our score was expected. The factors included in our risk score model, and the number of points these factors contributed to the risk score, highlight the need for patient monitoring, screening, and chronic disease prevention to minimise the risks of developing diabetes, hypertension, or CVD, as well as the risk of becoming hepatitis C virus coinfected.

The risk score model presented here establishes the demographic, traditional, and HIV-associated risk factors for CKD in those naïve to potentially nephrotoxic antiretrovirals. The antiretrovirals included in the model were chosen a priori based on previous research and include those suggested to have a potentially nephrotoxic effect [9,24]; we did not consider other antiretrovirals as they have not been shown to be associated with CKD. The potentially nephrotoxic antiretrovirals were then added as time-updated variables to estimate the increased risk of CKD after starting these antiretrovirals during follow-up, after adjustment for relevant risk factors for CKD. In addition, we developed a short version of the risk score excluding cardiovascular information—not always routinely available in HIV cohorts—which showed good agreement with the full risk score and also good agreement with external validation. The short version of the risk score could be widely used to assess renal risk in developing countries. The DART trial reported 5% of individuals with HIV developing CKD at 4 y, higher than the 2.7% overall found in the D:A:D study, and concluded that first-line ART, including tenofovir, could be used without renal monitoring [32]. The risk score developed here found that the risk of CKD was very low for the majority of HIV-positive individuals, and hence the NNTH from adding potentially nephrotoxic antiretrovirals was high, confirming the results from the DART trial, which primarily included individuals at low risk of CKD, and suggesting that such antiretrovirals can be started with minimal additional risk of CKD, subject to appropriate monitoring. In the high risk group (risk score ≥ 5), the NNTH was much lower, and routine renal monitoring in such patients, or the use of an alternative non-nephrotoxic antiretroviral if available, seems warranted in such high risk populations.

We validated both the full risk score and the short version, excluding important cardiovascular risk factors, on quite diverse populations. The Royal Free Hospital Clinic Cohort is a clinic-based cohort with a lower proportion of older individuals or intravenous drug users, while individuals from the randomised SMART/ESPRIT trials were from many centres worldwide [27,28]. As stated, we cannot adjust fully for race in the D:A:D study, and so we used the Cockcroft-Gault formula for estimating eGFR [22], adjusted for body surface area. Research from EuroSIDA has shown that eGFRs derived from the Cockcroft-Gault and CKD-EPI formulae predict CKD equally well in European patients [33]. The CKD-EPI formula was used in the validation cohorts to demonstrate that the risk score had good discrimination and was not sensitive to the choice of formula for calculating eGFR. Despite differences between the validation cohorts and the derivation cohort in frequency of and method for calculation of eGFR, there was remarkable agreement between the different studies in the increased incidence of CKD associated with a unit increase in the risk score and in the incidence rates of CKD within each risk score group, and excellent discrimination between those developing and not developing CKD.

There are several limitations to this study; we excluded over half of the D:A:D study population in order to determine risk factors for CKD in individuals naïve to potentially nephrotoxic antiretrovirals and to determine the subsequent medium-term increased risk of CKD when individuals at high, medium, or low risk of CKD start such drugs. Limitations on data collection meant we were not able to fully adjust for race, and further validation in this subgroup should be considered. Unmeasured confounding can never be excluded in observational studies. Our model considered only antiretrovirals previously suggested to have a nephrotoxic effect (tenofovir, atazanavir/ritonavir, and boosted protease inhibitors excluding lopinavir/ritonavir), and we cannot exclude the possibility that other antiretrovirals not included in these analyses may also increase the risk of CKD. Routine screening for proteinuria in HIV-positive individuals, which is associated with CKD in HIV infection, has only recently been implemented, and proteinuria screening data are not yet available in the D:A:D study and could not be included in our risk score model. We encourage other cohorts with available data to determine whether including such information improves the predictive ability of the D:A:D risk score, as well as to further develop the risk score to incorporate other prognostic information for CKD. A recently derived risk score for CKD in HIV-positive men, including proteinuria, suggested that similar clinical and laboratory markers were associated with CKD. Importantly, this study considered the impact of only tenofovir [19] and not other potentially nephrotoxic antiretrovirals, and was not externally validated

The major strength of the risk score presented here is that it provides a clinically useful, easily calculable, and implementable measure of future risk of CKD in HIV-positive individuals that facilitates an individualised approach to management by allowing calculation of the risk of CKD for a given set of characteristics, and then investigation of how that risk changes if potentially nephrotoxic antiretrovirals are added to the treatment regimen. The tool is available at http://hivpv.org/Home/Tools/ChronicKidneyDiseaseTool.aspx. The 2013 revised WHO guidelines recommend targeted creatinine testing for HIV-positive individuals taking tenofovir and for those with hypertension [34]. CKD is a multifactorial process, and resources might be better utilised by more frequent or targeted testing in those with a high risk score, which incorporates many different risk factors. The NNTH shows that adding potentially nephrotoxic antiretrovirals to the treatment regimen for patients at low underlying risk of CKD should not cause undue concern and that the benefits likely outweigh the risks. Conversely, the use of such antiretrovirals in individuals with a high underlying risk of CKD needs careful discussion and close monitoring. Risks and benefits, both renal and for other comorbidities, and already acquired HIV drug resistance clearly need to be carefully assessed when choice of antiretroviral drugs and intensity of monitoring is decided on in this group of individuals. The shorter version of the risk score can be widely implemented, even in settings where cardiovascular risk factors are unknown. Finally, our validation study in two diverse external cohorts showed that an increased risk score was associated with a very similar increased incidence of CKD in the derivation and validation cohorts.

In conclusion, we have developed and externally validated a risk score for CKD in HIV infection that has direct clinical relevance for patients and clinicians to weigh the benefits of certain antiretrovirals against the risk of CKD, and to identify those at greatest risk of CKD.

## Supporting Information

S1 Table Characteristics of validation cohorts. (DOCX)

## Abbreviations

aIRR: adjusted incidence rate ratio
ART: antiretroviral therapy
cART: combination antiretroviral therapy
CKD: chronic kidney disease
CKD-EPI: Chronic Kidney Disease Epidemiology Collaboration
CVD: cardiovascular disease
D:A:D: Data Collection on Adverse Events of Anti-HIV Drugs
eGFR: estimated glomerular filtration rate
IQR: interquartile range
NNTH: number needed to harm
PYFU: person-years of follow-up
