# Supplementary material for: Development and Validation of a Risk Score for Chronic Kidney Disease in HIV Infection Using Prospective Cohort Data from the D:A:D Study
Source: PLoS Med. 2015 Mar 31;12(3):e1001809. doi: 10.1371/journal.pmed.1001809 (PMC4380415; doi:10.1371/journal.pmed.1001809)
Supplement: S1 Table — (DOCX) [file pmed.1001809.s001.docx]

S1_Table : Characteristics of validation cohorts

|  |  | RFH | | SMART/ESPRIT | |
| --- | --- | --- | --- | --- | --- |
|  |  | N | % | N | % |
| N |  | 2548 | 100 | 2013 | 100 |
| Gender | Male | 1945 | 76.3 | 1523 | 75.7 |
|  | Female | 603 | 23.7 | 490 | 24.3 |
| HIV Risk | Non-IDU | 2472 | 97.0 | 1793 | 89.1 |
|  | IDU | 76 | 3.0 | 220 | 10.9 |
| Hepatitis C | No | 2405 | 94.4 | 1743 | 86.6 |
| Coinfected | Yes | 143 | 5.6 | 270 | 13.4 |
| Age | Median (IQR) | 36 | (31 – 42) | 42 | (35 – 49) |
|  | <35 | 1128 | 44.3 | 517 | 25.7 |
|  | >35 to <50 | 1216 | 47.7 | 1099 | 54.6 |
|  | >50 to < 60 | 164 | 6.4 | 313 | 15.6 |
|  | >60 | 40 | 1.6 | 84 | 4.2 |
| eGFR | Median (IQR) | 105 | (95 – 115) | 112 | (101 – 122) |
|  | >60 to <70 | 48 | 1.9 | 38 | 1.9 |
|  | >70 to <90 | 366 | 14.4 | 191 | 9.5 |
|  | >90 | 2134 | 83.7 | 1784 | 88.6 |
| Nadir CD4 | Median (IQR) | 298 | (142 – 481) | 238 | (130 – 350) |
|  | <200/mm^3^ | 869 | 34.1 | 840 | 41.7 |
|  | >200/mm^3^ | 1679 | 65.9 | 1173 | 58.3 |
| Baseline CD4 | Median (IQR) | 413 | (249 – 591) | 513 | (405 – 685) |
| Baseline (month/year) | Median (IQR) | 6/02 | (4/00 – 9/05) | 10/02 | (12/01 – 2/04) |
| Prior CVD |  |  |  | 64 | 3.2 |
| Prior diabetes |  |  |  | 98 | 4.9 |
| Prior hypertension |  |  |  | 437 | 21.7 |
| *During follow-up* |  |  |  |  |  |
| eGFRs | Median (IQR) | 22 | (11 – 34) | 6 | (4 – 7) |
| Months between eGFRs | Median (IQR) | 3.2 | (2.2 – 4.4) | 11.7 | (9.6 – 12.6) |

IQR; interquartile range. IDU’ intravenous drug user. eGFR calculated using CKD-EPI formula(28). CVD; cardiovascular disease. Hyp; hypertension.

Prior CVD in ESPRIT/SMART was defined as a stroke, myocardial infarction, coronary artery disease surgery or treatment at randomisation. Prior diabetes in ESPRIT/SMART was defined as the diagnosis of diabetes or the use if treatment for diabetes at randomisation. Prior hypertension in ESPRIT/SMART was defined as the use of blood pressure lowering treatment at randomisation or the use of lipid lowering treatment at randomisation
